# Interferon-α-Enhanced CD100/Plexin-B1/B2 Interactions Promote Natural Killer Cell Functions in Patients with Chronic Hepatitis C Virus Infection

**DOI:** 10.3389/fimmu.2017.01435

**Published:** 2017-11-03

**Authors:** Yu He, Yonghong Guo, Chao Fan, Yingfeng Lei, Yun Zhou, Mingjie Zhang, Chuantao Ye, Guangxi Ji, Li Ma, Jianqi Lian, Jonathan P. Moorman, Zhi Q. Yao, Jiuping Wang, Chunqiu Hao, Ying Zhang, Zhansheng Jia

**Affiliations:** ^1^Department of Infectious Diseases, Tangdu Hospital, Fourth Military Medical University, Xi’an, China; ^2^Department of Microbiology, The Fourth Military Medical University, Xi’an, China; ^3^HANK Biological Engineering Research Institute, Shenzhen, China; ^4^Department of Internal Medicine, Division of Infectious Diseases, James H. Quillen College of Medicine, Center of Excellence in Inflammation, Infectious Diseases, and Immunity, East Tennessee State University, Johnson City, TN, United States

**Keywords:** hepatitis C virus, natural killer cells, interferon-α, CD100, plexin-B1/B2

## Abstract

**Background:**

CD100, also known as Sema4D, is an immune semaphorin constitutively expressed on natural killer (NK) cells and T cells. As an immune activation molecule, CD100 has important immunoregulatory effects on NK functions by enhancing the interactions between NK cells and target cells. The aim of this study was to investigate whether hepatitis C virus (HCV) infection affects CD100 expression, and whether interferon-α treatment enhances NK killing activity to facilitate HCV clearance *via* CD100.

**Methods:**

Expression of CD100 on NK cells was evaluated by flow cytometry in patients with chronic HCV infection, with or without pegylated interferon-α-based therapy. NK cell cytotoxicity and interferon (IFN)-γ production were measured by flow cytometry upon culturing the NK cells with K562 and Huh7.5 or HCV JFH-1-infected Huh7.5 cells.

**Results:**

The frequency of CD100^+^ NK cells in HCV-infected individuals was slightly suppressed compared to healthy subjects. IFN-α treatment could significantly upregulate CD100 expression, which was confirmed by *in vitro* studies using peripheral blood mononuclear cells cocultured with HCV-expressing Huh7.5 cells or IFN-α. Importantly, the expression of CD100 on NK cells from HCV patients was inversely associated with the HCV-RNA levels in the early phase of IFN-α therapy, and the IFN-α upregulated CD100 led to an enhanced NK killing activity through ligations with its receptors plexin-B1/B2 on target cells.

**Conclusion:**

These results implied a novel mechanism by which IFN-α enhanced CD100/Plexin-B1/B2 interaction plays an important role in promoting NK functions in patients with chronic hepatitis C.

## Introduction

Hepatitis C virus (HCV) is a blood-borne pathogen, leading to severe liver diseases in millions of people worldwide (1). Immune responses play a crucial role in infection control and disease progression (2). Natural killer (NK) cells, an important innate immune cell population, provide early defense against viral infections by killing infected cells and producing cytokines, such as interferon (IFN)-γ, that inhibit viral replication (3). Recently, several studies have shown that NK cells are involved in anti-HCV immune responses in both acute and chronic HCV infection (4–9). Despite controversy regarding NK cell phenotype and function (8–12), NK cells are known to be functionally impaired during HCV infection (13, 14); the underlying mechanisms, however, have not been well defined.

While the current therapy for chronic hepatitis C involves direct-acting antiviral (DAA) agents, pegylated interferon (Peg-IFN)-α therapy provides a specific model for immune regulation during antiviral treatment (15). Therefore, it is important to understand the mechanisms of IFN-α-mediated HCV clearance. In addition to a direct antiviral activity (16), IFN-α likely exerts immunomodulatory effects on eliminating HCV-infected hepatocytes (17). Several studies have revealed that IFN-α-induced tumor necrosis factor-related apoptosis inducing ligand (TRAIL) and degranulation by NK cells play important roles in killing virus-infected cells (4, 8, 17). However, the potential molecular mechanisms underlying IFN-α-mediated anti-HCV immune responses remain elusive.

Semaphorins were originally identified as axon guidance factors involved in the development of the neuronal system. Sema4D, also known as CD100, was the first immune semaphorin discovered and is constitutively expressed on resting T cells and NK cells (18). CD72, plexin-B1, and plexin-B2 serve as the CD100 receptors in the immune system (19–21). Accumulating evidence indicates that CD100 plays an essential role in immune regulation by enhancing effector functions (20, 22–28).

Natural killer cell activation is primarily regulated by integration of signals from a diverse array of activating and inhibitory receptors (29, 30). The recognition and interactions between effectors and their targets exert essential effects on killing virus-infected cells. It has been reported that CD100, as an immune activation molecule, is involved in the NK killing process through signal activation between NK cells and target cells by binding to CD72 (31). Recently, Eriksson et al. have revealed that CD8^+^ T cells lacking CD100 expression are increased and functionally impaired during HIV infection (28), suggesting that viral infection might also have an impact on CD100 expression and its functions. In the study, we examined CD100 expression on NK cells from chronically HCV-infected patients with or without IFN-α therapy, including those with sustained virological response (SVR), and early virological response (EVR). The effects of CD100 on NK cell functions were also examined. For the first time, we demonstrate that CD100 expression is suppressed on NK cells in patients with chronic HCV infection, and IFN-α therapy can enhance CD100/plexin-B1/B2 interactions between NK cells and HCV-infected hepatocytes, leading to a more efficient killing activity and enhanced IFN-γ production by NK cells. These results suggest that IFN-α-enhanced CD100 expression may represent a novel mechanism involved in antiviral immune responses.

## Materials and Methods

### Subjects

This study includes the following subjects (as summarized in Table S1 in Supplementary Material): (i) 30 treatment-naive patients with chronic hepatitis C. All patients tested positive for anti-HCV with a third-generation enzyme-linked immunosorbent assay (Kechuang and Xinhua, Shanghai, China). HCV RNA levels were quantified using a reverse transcription polymerase chain reaction (RT-PCR) assay (Qiagen, Shenzhen, China), with a lower limit of detection for 100 copies/ml. All patients were negative for antibodies against hepatitis B, hepatitis D, and human immunodeficiency virus; (ii) 25 HCV patients who achieved an EVR (defined as serum HCV RNA being undetectable, <100 copies/ml), at week 12 after initiation of Peg-IFN-α-2a (180 μg/week subcutaneously) and weight-based ribavirin (800–1,200 mg, depending on the HCV genotype); (iii) 20 HCV patients who achieved an SVR (defined as HCV RNA remains undetectable for at least 6 months after treatment discontinuation) following Peg-IFN-α and ribavirin therapy; (iv) 24 age- and sex-matched healthy volunteers. Informed consent was obtained from all participating subjects. The study protocol conformed to the ethical guidelines of the 1975 Declaration of Helsinki and was approved by the Research and Ethical Committee of Tangdu Hospital of the Fourth Military Medical University.

### Cell Isolation and Purification

Peripheral blood mononuclear cells (PBMCs) were freshly isolated from peripheral blood by density gradient centrifugation using Ficoll-Hypaque (Sigma, St. Louis, MO, USA) and frozen or immediately studied as described below. If indicated, NK cells were further purified from PBMCs by negative selection using a human NK cell isolation kit (Miltenyi Biotec, Bergisch Gladbach, Germany) according to the manufacturer’s instructions. The purity of NK cells was measured by flow cytometry after staining the cells with anti-CD3-Cy5.5/PerCP (Clone: UCHT1, BD Biosciences, San Jose, CA, USA) and anti-CD56-Pe-Cy7 (Clone: B159, BD Biosciences, San Jose, CA, USA), with the frequency of CD3^−^CD56^+^ cells above 90% and CD3^+^ cells less than 1% to be used for subsequent experiments.

### HCVcc Generation and Infection

Hepatitis C virus JFH-1 (genotype 2a) was produced as described previously (32). The JFH-1 expression construct (kindly provided by Dr. C. Rice, Rockefeller University, New York, NY, USA) was linearized, and full-length JFH-1 RNA was transcribed using a MEGAscript T7 *in vitro* transcription kit (Ambion, Austin, TX, USA) per manufacturer’s instructions. 5 × 10^5^ Huh7.5 cells (kindly provided by Dr. C. Rice) were transfected at 70–80% confluent in a six-well plate with 2 μg transcribed RNA using DMRIE-C reagent per company’s protocol (Invitrogen, Carlsbad, CA, USA). HCV antigen expression was examined at 48 h after transfection by immunofluorescence using HCV NS5 antibodies (ViroGen, Watertown, MA, USA) and the supernatant collected from HCV RNA-transfected Huh7.5 cells at 48 h was used to infect naive Huh7.5 cells to make HCV stocks. HCV titer was detected as previously described (33).

Peripheral blood mononuclear cells (0.5 × 10^6^ cells) from healthy subjects were infected by coculture with JFH-1/Huh7.5 cells at an effector-to-target (E:T) ratio of 10:1 or with HCV particles at a multiplicity of infection (MOI) of 10 for 48 h. Complete cell culture medium was used as negative control. After incubation, cells were stained for anti-CD3 (Clone: UCHT1), CD14 (Clone: M5E2), CD19 (Clone: HIB19), CD56 (Clone: B159), CD16 (Clone: 3G8), all from BD Biosciences, San Jose, CA, USA, and CD100 (Clone: A8, BioLegend, San Diego, CA, USA) monoclonal antibodies followed by flow cytometric analysis.

### IFN-α Treatment

We also observed the effect of IFN-α on CD100 and plexin-B1/B2 expression. PBMCs (0.5 × 10^6^ cells) were incubated in 1 ml complete medium supplemented with IFN-α-2a (at a concentration ranging from 0.01 to 1,000 ng/ml) (Roche Bioscience, Hillview Avenue Palo Alto, CA, USA) in a 24-well round-bottom plate (Corning, One Riverfront Plaza, NY, USA.) for 2, 6, 12, 24, and 48 h, respectively, and Huh7.5 cells were stimulated with IFN-α-2a (10 ng/ml) for 48 h. Complete cell culture medium was used as controls. After incubation, cells were stained with monoclonal antibodies as described above for flow cytometric analysis.

### NK Cell Phenotypic and Functional Characterization

For phenotypic analysis, PBMCs isolated from HCV patients and healthy subjects were stained with CD3-Cy5.5/PerCP (Clone: UCHT1), CD14-Cy5.5/PerCP (Clone: M5E2), CD19-Cy5.5/PerCP (Clone: HIB19), CD16-Cy7/APC (Clone: 3G8), CD56-PeCy7 (Clone: B159), CD69-FITC (Clone: FN50), TRAIL-PE (Clone: S35-934), all from BD Biosciences, San Jose, CA, USA, CD100-FITC (Clone: A8, BioLegend, San Diego, CA, USA) or isotype matched controls (BD Biosciences, San Jose, CA, USA). Plexin-B1/B2 expression on K562 (ATCC, Manassas, VA, USA) and Huh7.5 cells (used as target cells) were measured using RT-PCR (primers were listed in Table S2 in Supplementary Material) and flow cytometry with plexin B1 and plexin B2 antibody (Clone: #559830&#537223, R&D Systems Inc., Minneapolis, MN, USA). Immunostained cells were analyzed on a multicolor Arial II (BD Biosciences, San Jose, CA, USA) and FlowJo Version 7.6.2 software.

For analysis of cytokine production and degranulation, purified NK cells were resuspended at 10^5^ cells/ml and stimulated with either (i) K562, Huh7.5 or JFH-1/Huh7.5 cells at an E:T ratio of 1:1; (ii) K562, Huh7.5 or JFH-1/Huh7.5 cells at an E:T ratio of 1:1 in the presence of IFN-α (10 ng/ml); (iii) K562, Huh7.5 or JFH-1/Huh7.5 cells preincubated for 2 h with soluble CD100 (sCD100) (PeproTech, Rocky Hill, NJ, USA) or antiplexin B1/2 antibodies mix (Clone: sc28372&sc373969, Santa Cruz, CA, USA) to block availability of plexin-B1/B2; (iv) complete cell culture medium without target cells and cytokines as control. After 8 h incubation at 37°C, Brefeldin A (10 ng/ml) (eBioscience, San Diego, CA, USA) and monensin (0.67 μl/ml) (BD Biosciences, San Jose, CA, USA) were added for another 4 h prior to harvesting the cells. Finally, NK cells were washed, stained with anti-CD3, CD16, CD56, and CD100, and analyzed by flow cytometry.

### RNA Extraction and Quantification

RNA was extracted using the RNeasy mini kit (Qiagen, Hilden, Germany) according to the manufacturer’s instructions. Quantification of messenger RNA (mRNA) levels of plexin-B1 and plexin-B2 were performed by real-time PCR. GAPDH served as the internal control. Primer sequences used for real-time PCR analysis were listed in Table S2 in Supplementary Material.

### Statistical Analysis

Statistical analyses were performed with GraphPad Prism Version 5.0 (GraphPad Software Inc., San Diego, CA, USA). The Shapiro–Wilk test was used to test the normal distribution of quantitative variables. When quantitative variables were normally distributed, the results were presented as mean ± SD, otherwise median and interquartile range (25th–75th percentile) were reported; differences between two or three groups were evaluated by parametric or non-parametric tests according to data distribution. Paired Student’s *t*-test was used to assess paired variables. Correlations between variables were evaluated with the Spearman rank correlation test. *p* < 0.05 was considered as statistically significant.

## Results

### Phenotypic Change of NK Cells from Chronic HCV Infection

**Figure 1:**
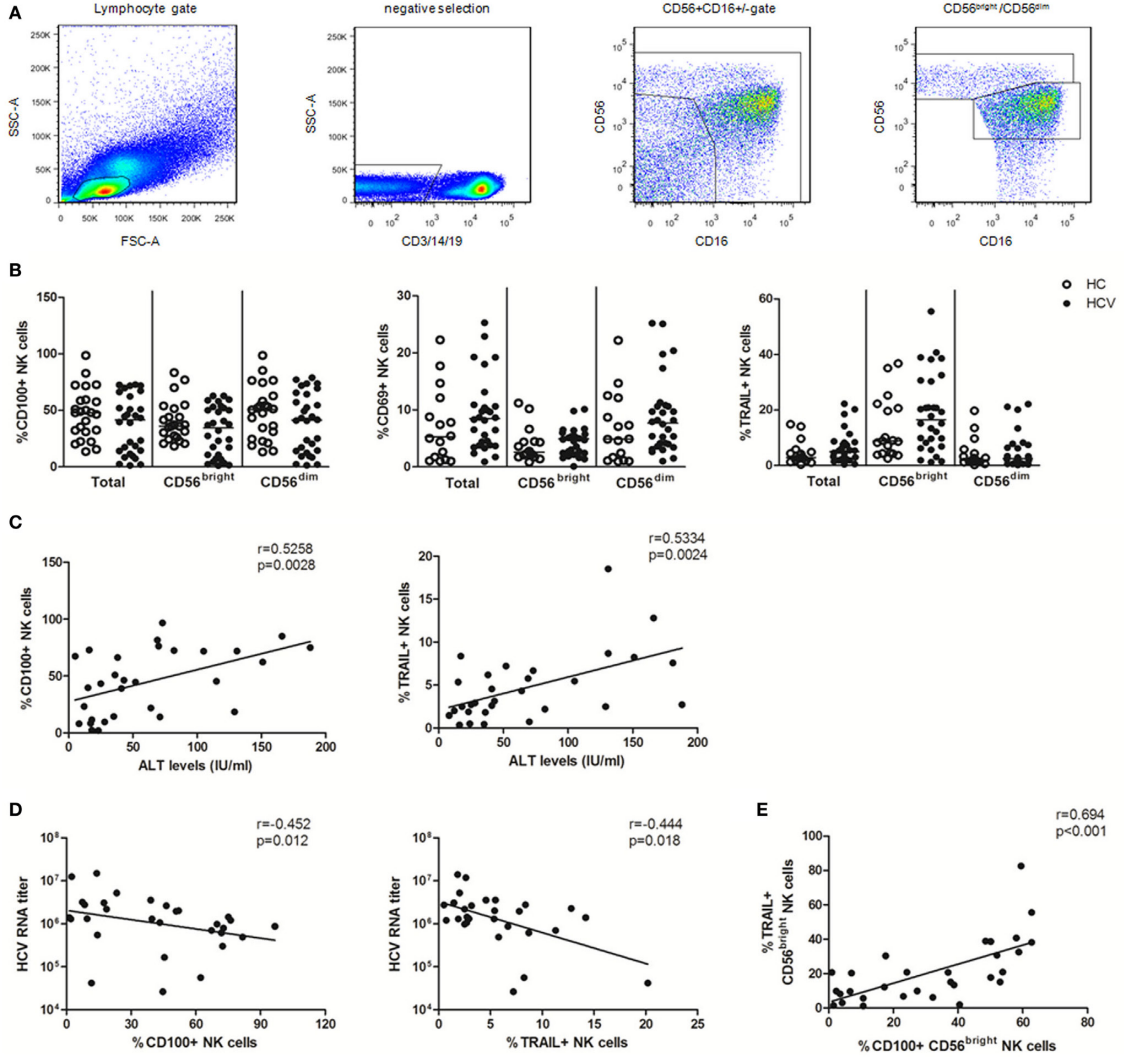
Natural killer (NK) cell phenotypic analysis in patients with chronic hepatitis C virus (HCV) infection. 99 subjects were recruited in the studies, including 24 healthy subjects, 30 HCV patients, 25 early virological response (EVR) patients, and 20 sustained virological response (SVR) patients. All HCV patients were treated with pegylated interferon (Peg-IFN)-α and RBV therapy. **(A)** Gating strategy. **(B)** Frequency of CD100, CD69, and TRAIL expression on total NK and two subsets in patients with chronic HCV infection (HCV, filled circles) and healthy subjects (HC, open circles). **(C,D)** Correlation analysis between CD100 or TRAIL expression on NK cells and ALT levels or HCV-RNA titer. **(E)** The relationship between CD100 and TRAIL in CD56^bright^ NK cells. The Mann–Whitney *U* test was used for the statistical analysis.

Based on CD56 and CD16 expression, NK cells can be divided into CD56^bright^ (CD56^++^CD16^±^) and CD56^dim^ (CD56^+^CD16^+^) subsets (34). CD3/14/19^−^CD56^+^ NK cells and their CD56^bright^ and CD56^dim^ subsets were identified in PBMCs using multicolor flow cytometry by immune-staining for CD3, CD14, CD19, CD56, and CD16 (Figure 1A). To identify the specific effect of HCV infection on NK cells, we conducted an *ex vivo* cross-sectional analysis to investigate the CD100, CD69, and TRAIL expression on NK cells from HCV patients versus healthy subjects. As shown in Figure 1B, the percentage of CD100^+^ NK cells was slightly decreased in patients with chronic HCV infection compared to healthy subjects, though this did not achieve a significant difference. Similarly, no significant changes were observed in the frequency of CD69^+^ NK and TRAIL^+^ NK cells between HCV patients and healthy subjects, though these phenotypic markers were slightly upregulated in the setting of HCV infection. These data suggest that NK cells were not successfully activated during chronic HCV infection.

To investigate whether CD100 was implicated in liver injury and disease progression, we observed the correlation between the measured molecules with ALT and HCV-RNA levels. We found that CD100^+^ NK as well as TRAIL^+^ NK cells positively correlated with the serum ALT levels, but inversely correlated with the HCV-RNA titers (Figures 1C,D). Moreover, the level of CD100 was found to be related to the TRAIL expression on CD56^bright^ NK cells (Figure 1E); however, no correlations were found between the CD69 expression on NK cells and the ALT or HCV-RNA levels.

### IFN-α Significantly Upregulated CD100 Expression on NK Cells

**Figure 2:**
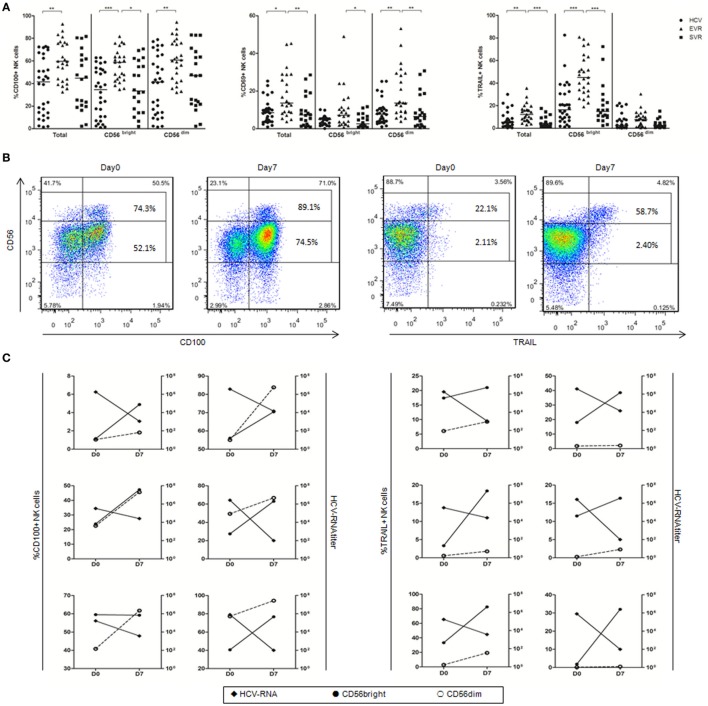
Alteration of natural killer (NK) cell phenotype in hepatitis C virus (HCV) patients after initiating antiviral treatment. **(A)** The percentage of CD100, CD69, and TRAIL expression on total NK and subsets in treatment-naive patients with chronic hepatitis C (circles), patients with early virological response (EVR, triangles) and sustained virological response (SVR, squares) after IFN-α-based therapy. **(B)** Representative staining of CD100 and TRAIL in one patient with the frequency of CD100 and TRAIL expressing CD56^bright^ and CD56^dim^ NK cells. **(C)** CD100 and TRAIL expression on CD56^bright^ (filled circles) and CD56^dim^ NK cells (open circles) and HCV-RNA titer (filled rhombus). *p < 0.05, **p < 0.01, ***p < 0.001. Dunn’s Multiple Comparison was used for comparison between the two groups. Kruskal–Wallis *H* was employed for comparison more than three groups. HCV patients were treated with Peg-IFN-α (180 μg/injection) and RBV (1,200 mg/d for genotype 1b; 900 mg/d for genotypes 2 and 3).

To determine whether IFN-α can activate NK cells *in vivo* and upregulate CD100 expression, we examined CD100, CD69 and TRAIL expressions by NK cells before and after IFN-α treatment in chronically HCV-infected patients who achieved EVR and SVR, respectively. We found that the proportion of CD100^+^, CD69^+^ and TRAIL^+^ NK cells was significantly upregulated in patients with EVR, and returned to normal in patients with SVR after IFN-α treatment (Figure 2A). Of note, the expression of CD100 was increased in both CD56^bright^ and CD56^dim^ NK subsets, whereas CD69 expression mainly increased in CD56^dim^ NKs, and TRAIL expression primarily increased in CD56^bright^ NK subsets, respectively (Figure 2A). Furthermore, we also examined the relationship between CD100 or TRAIL expression and HCV RNA titer in 6 patients at the very early phase of Peg-IFN-α and ribavirin treatment. PBMCs from these patients were collected on days 0 and 7 of treatment, and HCV-RNA in serum was quantified. At day 0 of treatment, about 74.3% of CD56^bright^ NKs and 52.1% of CD56^dim^ NKs were CD100^+^ cells. After 7 days of treatment, however, the percentage of CD100^+^ CD56^bright^ NKs and CD100^+^ CD56^dim^ NKs increased to 89.1 and 74.5%, respectively. Similarly, TRAIL^+^ cells were also increased from 22.1 to 58.7% in CD56^bright^ NK subset (Figure 2B). Importantly, the upregulation of CD100 on both NK subsets and TRAIL on CD56^bright^ NK cells were inversely correlated with the HCV-RNA decline during the early phase of antiviral treatment (Figure 2C). These results indicated that IFN-α treatment efficiently activated NK cells, and the increased CD100 and TRAIL expressions on NK cells correlated with the HCV control.

### IFN-α Increased CD100 and Plexin-B1/B2 Expressions *In Vitro*

**Figure 3:**
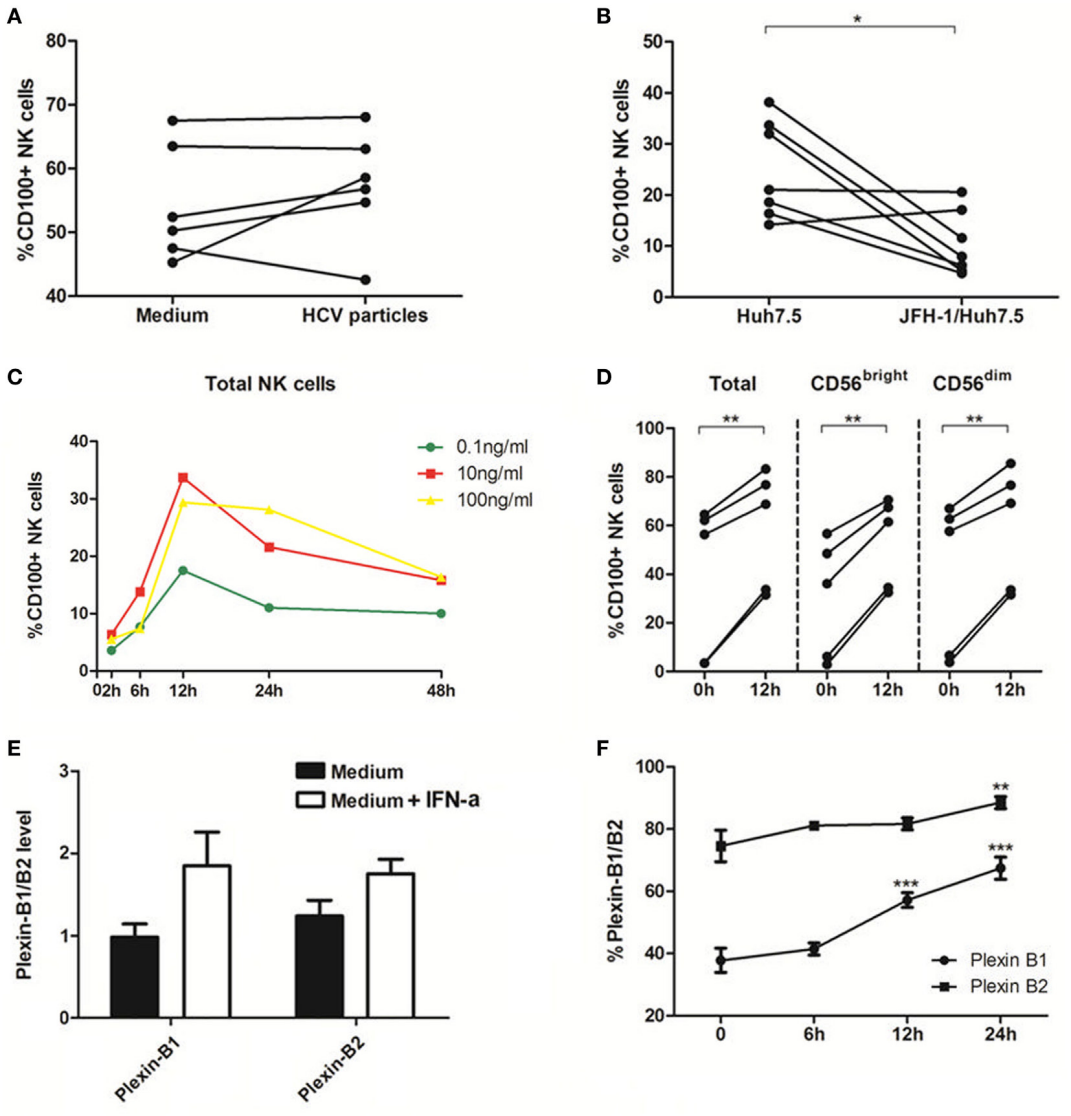
Interferon (IFN)-α increases CD100 and plexin-B1/B2 expression *in vitro*. **(A,B)** Peripheral blood mononuclear cells (PBMCs) cocultured with medium alone vs. medium containing hepatitis C virus (HCV) virions [multiplicity of infection (MOI) = 10]. **(C,D)** PBMCs cocultured with Huh7.5 cells vs. JFH-1/Huh7.5 cells. Huh7.5 cells were infected by the supernatant of HCV JFH-1-transfected Huh7.5 cells at 48 h, E:T = 10:1. **(E,F)** Plexin-B1 and plexin-B2 was upregulated by IFN-α (10 ng/mL) and the effect reached its maximum at 24 h after IFN-α treatment. *p < 0.05, **p < 0.01, ***p < 0.001. Paired Student’s *t*-test was used for the data analysis.

To further elucidate the role of HCV in regulation of CD100 expression and to mimic the *in vivo* setting of HCV infection, we employed a cell coculture system by incubating healthy PBMCs with Huh7.5 hepatocytes transfected with HCV JFH-1 strain *in vitro*. NS5 protein was detected in HCV-JFH-1 transfected Huh7.5 cells by immunofluorescent staining (Figure S1A in Supplementary Material). Additionally, Huh7.5 cells can be infected by the supernatant of JFH-1-transfected Huh7.5 cells (Figure S1B in Supplementary Material), suggesting that HCV particles are secreted from the HCV mRNA-transfected hepatocytes into the culture media. We incubated healthy PBMCs with medium alone vs. medium containing HCV virions (MOI = 10) or Huh7.5 vs. JFH-1-infected Huh7.5 cells for 48 h, followed by immune-staining and flow cytometric analysis. As shown in Figure 3A, CD100 expression on NK cells was not affected by the HCV particles; but it was remarkably decreased on PBMCs cocultured with JFH-1-infected Huh7.5 compared with those incubated with Huh7.5 cells without HCV infection (Figure 3B).

Next, we examined the effect of IFN-α on CD100 expression on NK cells, and found that CD100^+^ NK cells were significantly upregulated, in a dose- and time-dependent manner, in PBMCs stimulated with IFN-α (Figure 3C). CD100 expression began to rise at 6 h and peaked at 12 h following IFN-α treatment. As shown in Figure 3D, the same effect was observed in different NK subsets in PBMCs derived different donors incubated with IFN-α for 12 h. We also examined whether IFN-α can affect the expression of CD100 receptors, plexin-B1/B2, on Huh7.5 cells. Indeed, plexin-B1 and plexin-B2 expressions were upregulated by IFN-α, which reached a maximum effect at 24 h, following IFN-α treatment (Figures 3E,F). The CD72, another important CD100 receptor, was checked as well. However, it cannot be detected on Huh7.5 cells or HCV-infected/IFN-α treated Huh7.5 cells. These results indicated that IFN-α treatment can enhance CD100 and plexin-B1/B2 expressions on NK cells, which may influence signal transduction between NK cells and target cells.

### IFN-α Enhanced NK Cell Degranulation and IFN-γ Production

**Figure 4:**
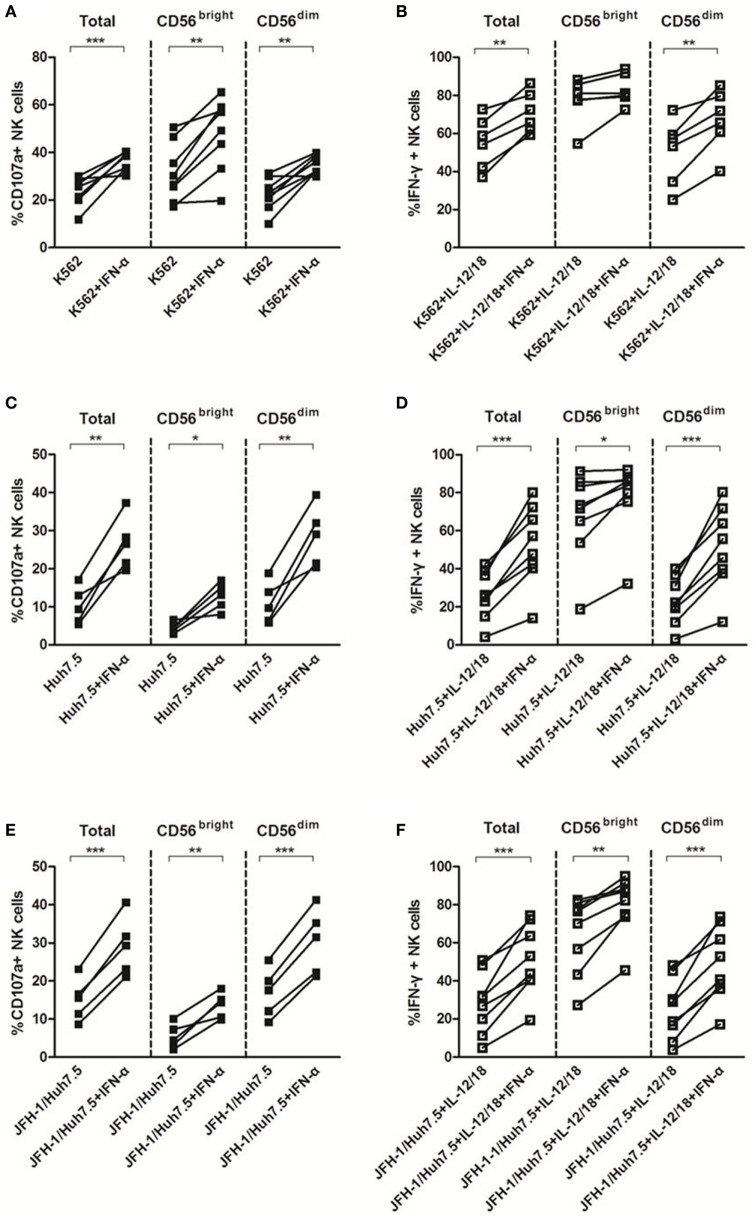
The effect of interferon (IFN)-α on CD107a and IFN-γ expressions by natural killer (NK) cells in response to target cells *in vitro*. Peripheral blood mononuclear cells (PBMCs) were incubated with medium containing hepatitis C virus (HCV) at multiplicity of infection (MOI) of 10 or Huh7.5 cells or JFH-1/Huh7.5 cells, with or without IFN-α treatment, in the presence of K562 target cells for 48 h, respectively. Huh7.5 cells were infected by the supernatant of HCV JFH-1-transfected Huh7.5 cells at 48 h, E:T = 10:1. **(A,C,E)** CD107a expression and **(B,D,F)** IFN-γ expression on total NK and NK subsets was measured by flow cytometry. *p < 0.05, **p < 0.01, ***p < 0.001. Paired Student’s *t*-test was used for the data analysis.

Natural killer cell activation typically results in cytotoxicity/degranulation and release of antiviral cytokines, including IFN-γ. Several lines of evidence have shown that NK cells from HCV-infected patients display a functional dichotomy, characterized by normal or enhanced cytotoxicity and reduced production of IFN-γ (8–10, 35, 36). To investigate whether HCV and IFN-α could affect NK cell killing activity to target cells in the *in vitro* system, we cocultured purified NK cells with K562, Huh7.5 or HCV JFH-1-infected Huh7.5 (JFH-1/Huh7.5) cells in the presence or absence of IFN-α. As shown in Figure S1C in Supplementary Material, HCV had a strong effect on CD107a expression, especially on CD56^dim^ subsets, but not on IFN-γ production (Figure S1D in Supplementary Material). In response to K562 cells, IFN-α treatment effectively activated NK cells through increased degranulation on both NK subsets and enhanced IFN-γ production on CD56^dim^ NK subset (Figures 4A,B). Correspondingly, similar results were observed (increased CD107 and IFN-γ expression in NK cells, particularly in CD56^dim^ subsets) upon coculturing NK cells with Huh7.5 (Figures 4C,D) or JFH-1/Huh7.5 cells (Figures 4E,F).

### CD100/Plexin-B Interactions Involved in NK Functions in Response to Target Cells

**Figure 5:**
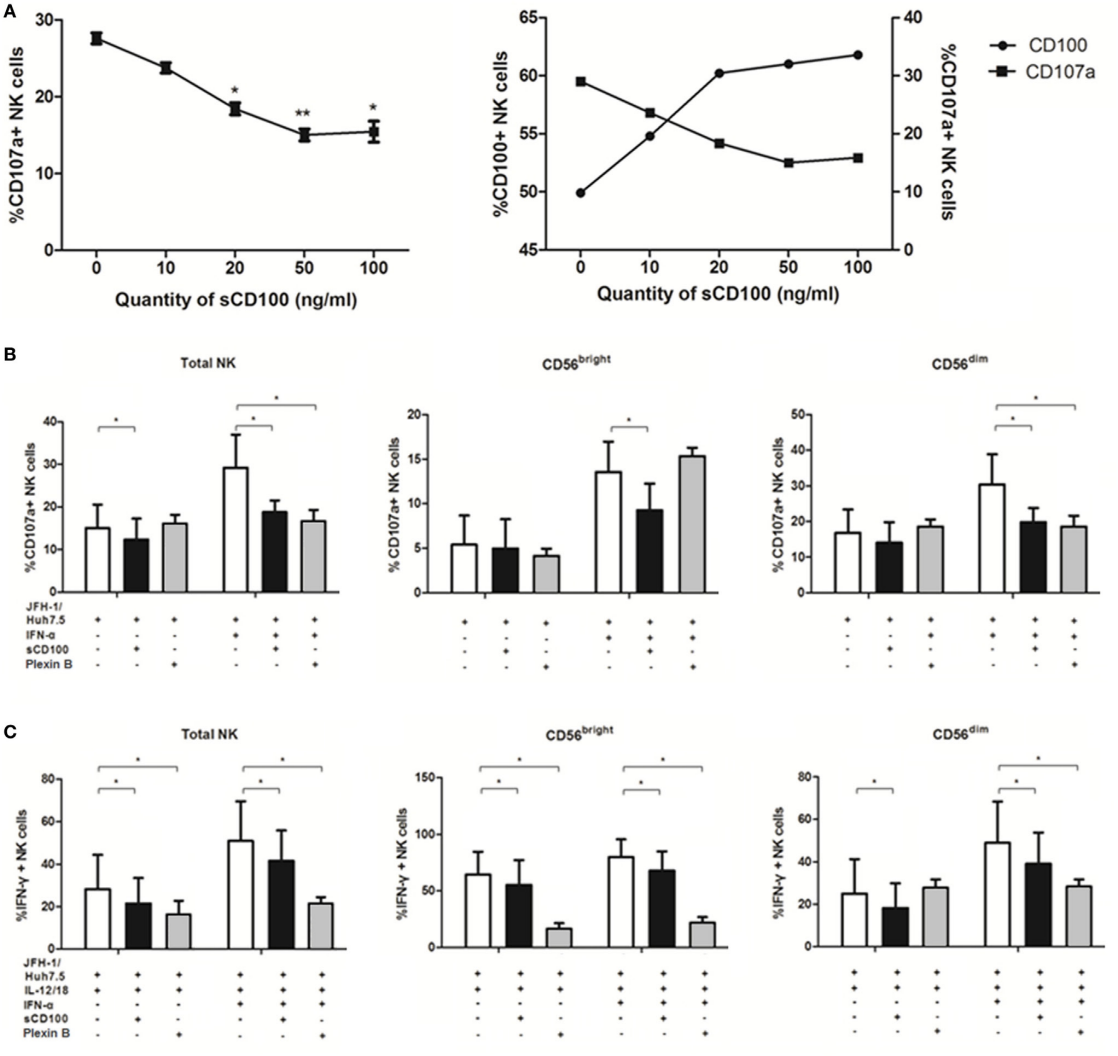
CD100–plexin-B1/B2 interactions involved in natural killer (NK) cell killing process. CD100–plexin-B1/B2 interactions were blocked by sCD100 or plexin B1/B2 antibodies preincubating. **(A)** Dose-dependent effect of sCD100 on the expressions of CD107a and CD100 on NK cells in the context of interferon (IFN)-α treatment. **(B,C)** CD107a and IFN-γ expressions in total NK and the two subsets, in the presence or absence of IFN-α, sCD100, or plexin B1/2 antibodies. *p < 0.05, **p < 0.01, ***p < 0.001. Paired Student’s *t*-test was used for the data analysis.

Previous studies have reported that degranulation and IFN-γ production of NK cells could be induced *via* activation of the IFN-α/β receptor (37, 38), it was not clear however whether other molecules were involved in this process, especially in response to target cells. To explore the mechanisms underlying NK cell functions in response to target cells, we studied the potential involvement of IFN-α, CD100, and plexin-B1/B2 in NK degranulation and IFN-γ production. Since we have shown that IFN-α is involved in NK regulation, we hypothesized that CD100 and plexin-B1/B2 interactions have functional consequences on the NK killing process. To test this hypothesis, we blocked the availability of plexin-B1/B2 by preincubating sCD100 with K562 cells. As shown in Figure 5A, the optimal blockade was observed at a concentration of 50 ng/ml, with a plateau being observed thereafter. Interestingly, it was found that the expression of CD107a on NK cells declined, whereas CD100 expression upregulated with increasing the sCD100 concentrations. This phenomenon might imply a negative loop between CD100–plexin-B interaction and CD100 expression. CD100–plexin B ligation could trigger Erk signaling within the cells (20). Thus, we believe that the ligations of CD100–plexin-B downregulate CD100 expression on NK cells through the MAPK signal transduction, whereas the interaction blockage by sCD100 rescues CD100 expression in NK cells. The plexin B1/2 antibodies were also used for CD100/plexin B interaction blockade according to previous studies (39). As expected, CD107a expression decreased in total NK as well as in the two NK subsets, particularly in the presence IFN-α after blocking plexin-B1/B2 availability by sCD100 or plexin B1/2 antibodies (Figure 5B); this was also true for IFN-γ production (Figure 5C). Taken together, these results revealed an important role of CD100–plexin-B1/B2 interactions in NK degranulation and IFN-γ production in response to target cells.

## Discussion

**Figure 6:**
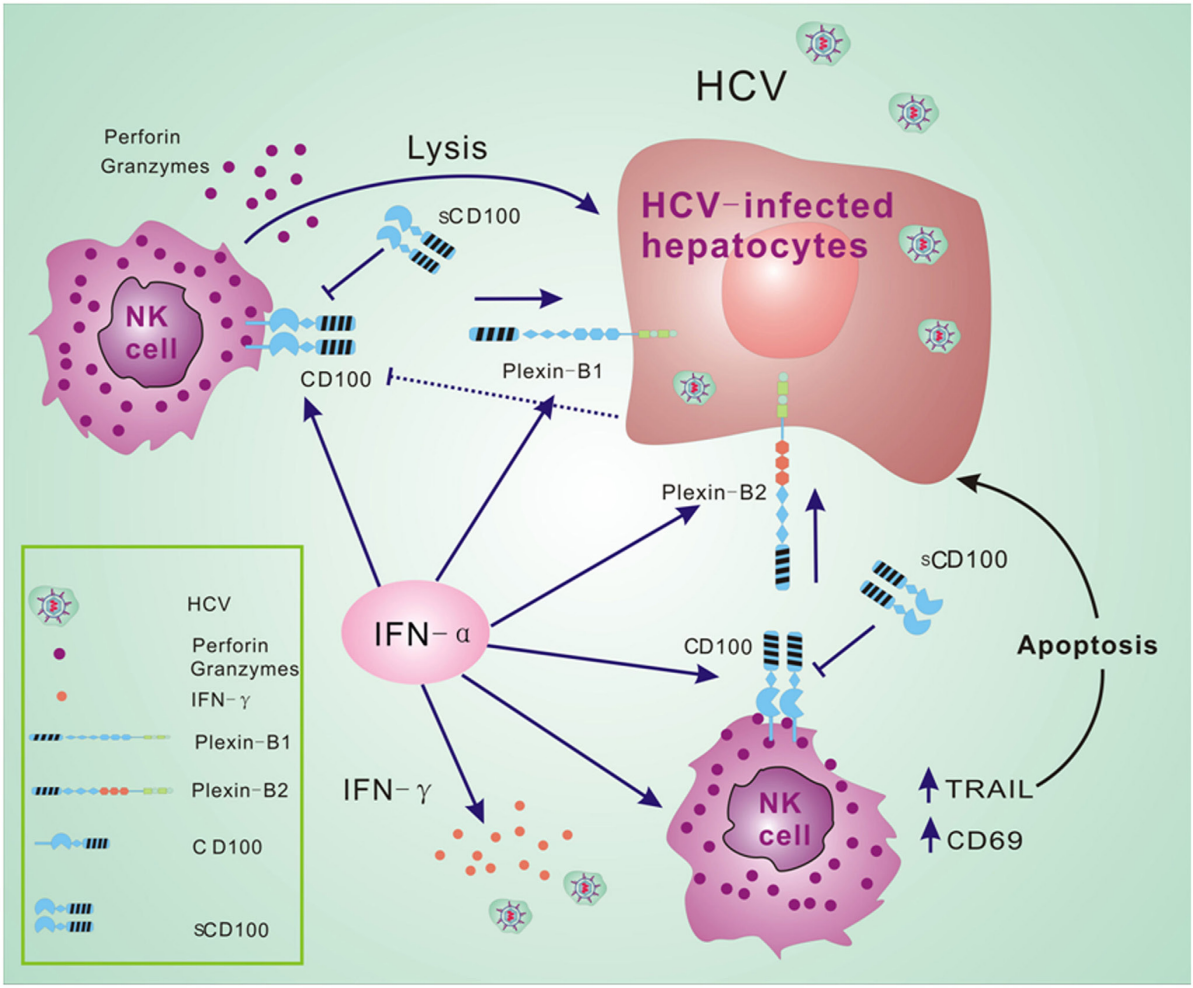
CD100–plexin-B1/B2 interactions facilitate immune responses of natural killer (NK) cells. We propose a model that NK cells recognize hepatitis C virus (HCV)-infected hepatocytes *via* CD100–plexin-B1/B2 interactions and facilitate the NK killing activity in HCV infection. Interferon (IFN)-α-based therapy not only efficiently activates NK cells, but improves the activation between NK effectors and HCV-infected cells *via* increased CD100 and plexin-B1/B2 expressions.

Many molecules are involved in the complex immune response that occurs during HCV infection and antiviral treatment. Our findings in this study suggest that HCV infection and IFN-α treatment affect the expressions of CD100 and its receptors, plexin-B1/B2. IFN-α further upregulates CD107a and IFN-γ expression in NK cells in response to target cells. Based on the novel finding of CD100 changes in HCV patients before and after antiviral treatment, we believe that the CD100–plexin-B1/B2 interactions play an important role in NK cell killing activity in response to HCV infection. We thus propose a model that in the HCV-infected liver, NK cells recognize HCV-infected hepatocytes *via* CD100–plexin-B1/B2 interactions so as to facilitate NK killing activities. IFN-α-based therapy not only efficiently activates NK cells, but improves the interactions between NK effectors and HCV-infected cells *via* increases in CD100 and plexin-B1/B2 expressions (Figure 6).

Natural killer cell activity is regulated through three major mechanisms: first, the balance between inhibitory and activating receptors on NK cell surface; second, the cross-talk with other cells, especially with dendritic cells (40, 41); and third, NK cell recognition and interaction with virus-infected cells (31, 42). Here, we demonstrate that HCV infection does not completely activate NK cells; i.e., the expressions of CD100, the early activation marker CD69, and the apoptotic molecule TRAIL in our HCV cohort are found at similar levels to those observed in healthy subjects. Earlier reports revealed that NK cell activity was compromised after exposure to HCV virions or HCV-infected hepatocytes (13, 14, 32, 43, 44). Consistent with two of these studies (32, 43), CD100 expression on NK cells is decreased after exposure to JFH-1/Huh7.5 cells, but not to HCV particles in our *in vitro* experiments.

Partially in line with other reports (4, 8, 17, 36), we demonstrate that IFN-α significantly upregulates the expression level of CD100, CD69, and TRAIL in chronic HCV patients after initiation of antiviral treatment, and this is also supported by our *in vitro* IFN-α stimulation assays. Furthermore, CD100 and TRAIL expression on NK cells appears to correlate with HCV clearance. To mimic the *in vivo* setting, we used PBMCs derived from HCV patients to examine CD100 expression on NK cells by IFN-α stimulation. Notably, CD100 can regulate many immune responses through CD72- or plexin B-induced downstream signal activation; however, the upstream elements of CD100 regulation remain unclear. Based our previous results, IFN-α positively regulates CD100 expression. And it is likely through control of JAK-STAT signal transduction (45, 46). On the other hand, our results demonstrate that HCV infection suppresses CD100 expression on NK cells. In conjunction with HCV’s suppressive effects on JAK-STAT signaling (47, 48), these data indirectly support the notion that the JAK-STAT pathway may interface with CD100 expression on NK cells.

Based on the correlation analysis, we speculate that CD100 and TRAIL might be associated with immune-mediated hepatic inflammation and elimination of HCV infection. Moreover, CD100 expression is related to TRAIL expression in CD56^bright^ NK cells, further suggesting that CD100 is associated with TRAIL-mediated apoptosis of HCV-infected cells. Interestingly, while the level of CD100 is related to TRAIL expression on CD56^bright^ NK cells, it is not on CD56^dim^ NK cells. CD100 exerts different effect on CD56^bright^ and CD56^dim^ NK subsets, implying different signal transductions exist between the NK subsets. As such, alteration of expression of CD100 and its receptors plexin-B1/B2 could be a potential antiviral mechanism for IFN-α treatment.

Several studies have demonstrated that IFN-α treatment markedly increases CD107a expression and decreases IFN-γ production (4, 6, 8, 9, 36), and work by Szabo indicates that IFN-γ production is increased after exposure to exogenous IFN-α (41). As with these investigations, we also employed HCV-infected Huh7.5 as target cells to investigate variations in NK functions. Previous studies have reported that CD100, which is significantly upregulated after cellular activation (21, 26, 49), and has an immunomodulatory effect on both humoral and cellular immune responses (18, 20, 22, 25, 27, 50). Recently, Eriksson et al demonstrated that CD100 is also involved in T cell responses during HIV infection (28). However, it is still unclear whether chronic HCV infection affects CD100 expression on NK cells and related immune responses.

We reveal a novel mechanism in which NK cell degranulation and IFN-γ production are enhanced through CD100–plexin-B1/B2 interactions between NK effectors and target cells. We also demonstrate that IFN-α can improve the NK killing process by increasing the expression of CD100 and its receptors plexin-B1/B2. To elucidate the underlying mechanisms, we performed *in vitro* experiments to block plexin-B1/B2 availability, preventing CD100–plexin-B1/B2 ligation. Our results demonstrate that CD100–plexin-B1/B2 interactions participate in NK degranulation and IFN-γ production, which are functional during IFN-α treatment.

We also show that CD100 is related to TRAIL expression on CD56^bright^ NK cells. TRAIL-triggered death pathway signaling appears to be an important mechanism for elimination of HCV-infected hepatocytes (4, 8, 17, 51). In line with this, we speculate that CD100–plexin-B1/B2 ligations between NK effector cells and target cells may facilitate TRAIL-mediated apoptosis.

In conclusion, we show a unique mechanism whereby IFN-α-based treatment enhances NK functions *via* increased CD100–plexin-B1/B2 ligations between NK and target cells, which may play an important role in HCV infection and antiviral treatment.

## Ethics Statement

Study protocol was approved by the Research and Ethical Committee of Tangdu Hospital, Fourth Military Medical University. Study was performed in accordance with the principles of Helsinki Declaration. Written informed consent was received from all the participants.

## Author Contributions

YH, YG, and CF: collection of patient material, data collection, acquisition, analysis and interpretation, statistical analysis, and manuscript writing. YL, YZhou, MZ, CY, GJ, LM, JL, JPM, ZQY, JW, and CH: revision of the manuscript. YZhang and ZJ: study concept and design, selection of samples and critical revision of manuscript.

## Conflict of Interest Statement

The authors declare that the research was conducted in the absence of any commercial or financial relationships that could be construed as a potential conflict of interest.

## Supplementary Material

Figure S1 NS5 protein was detected in HCV-JFH-1 transfected Huh7.5 hepatocytes by immunofluorescent staining **(A)**. Additionally, Huh7.5 cells can be infected by the supernatant of JFH-1-transfected Huh7.5 cells **(B)**. Purified NK cells were cocultured with K562, Huh7.5 or HCV JFH-1infected Huh7.5 (JFH-1/Huh7.5) cells in the presence or absence of IFN-α. It is showed that HCV had a strong effect on CD107a expression **(C)**, especially on CD56^dim^ subsets, but not IFN-γ production **(D)**.

Figure S2 Frequency of CD100, CD69 and TRAIL expression on total NK and two subsets in patients with chronic HCV infection and healthy subjects, analyzed by flow cytometry.

Figure S3 The percentage of CD100, CD69 and TRAIL expression on total NK and subsets in treatment-naive patients with chronic hepatitis C, patients with EVR and SVR after IFN-α-based therapy, analyzed by flow cytometry.

Figure S4 CD100-Plexin-B signaling influences NK cell functions. CD100-Plexin-B1/B2 interactions between NK and K562/Huh7.5 cells were blocked by sCD100 pre-incubating. CD107a and IFN-γ expressions were measured in total NK and the two subsets (CD56bright and CD56dim), in the presence or absence of IFN-α or sCD100. Paired Student t-test was used for the data analysis.
