# Supplementary figures and images for: Interferon-α-Enhanced CD100/Plexin-B1/B2 Interactions Promote Natural Killer Cell Functions in Patients with Chronic Hepatitis C Virus Infection
Source: Front Immunol. 2017 Nov 3;8:1435. doi: 10.3389/fimmu.2017.01435 (PMC5676449; doi:10.3389/fimmu.2017.01435)

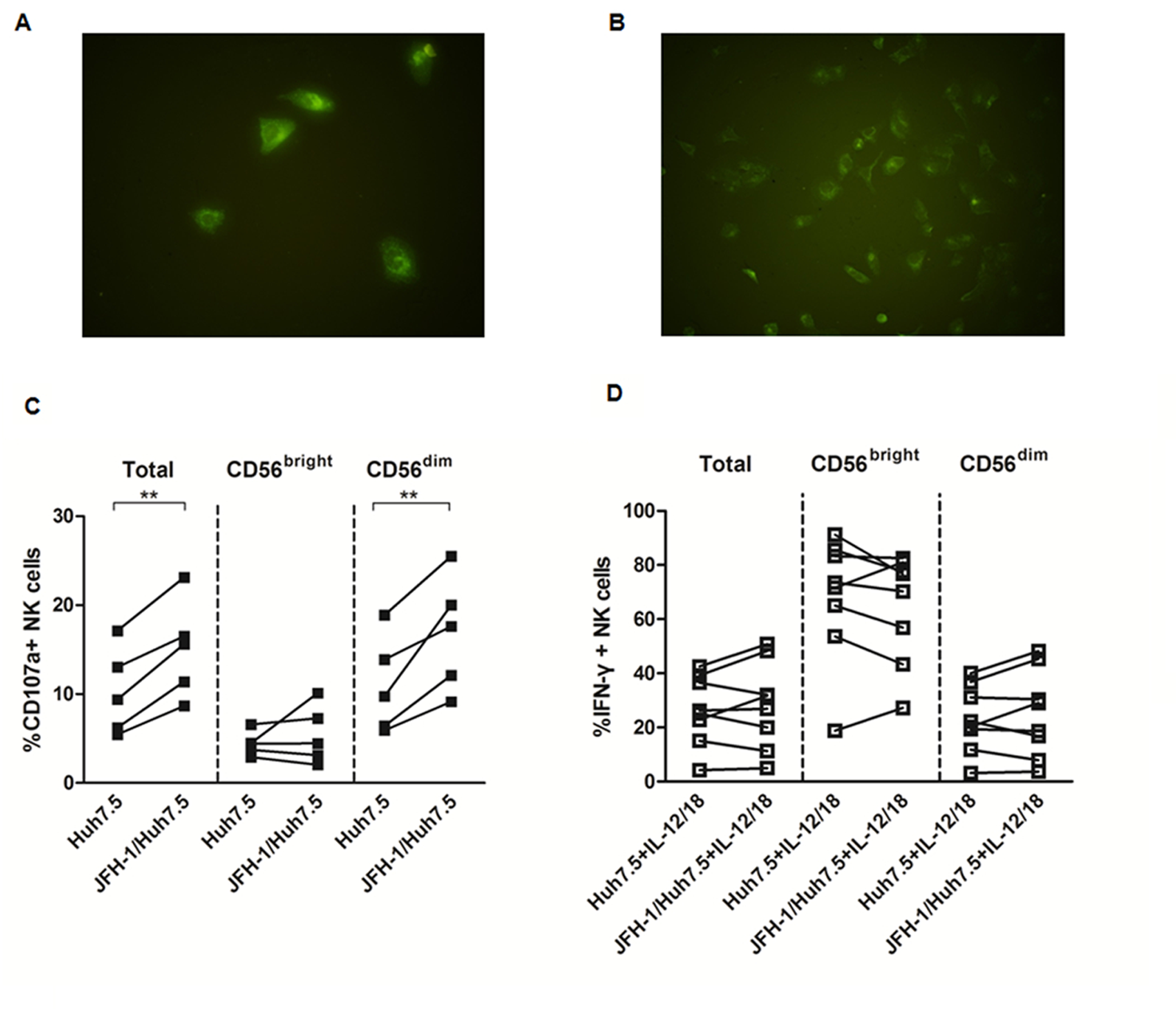

Supplement: Figure S1 — NS5 protein was detected in HCV-JFH-1 transfected Huh7.5 hepatocytes by immunofluorescent staining (A). Additionally, Huh7.5 cells can be infected by the supernatant of JFH-1-transfected Huh7.5 cells (B). Purified NK cells were cocultured with K562, Huh7.5 or HCV JFH-1infected Huh7.5 (JFH-1/Huh7.5) cells in the presence or absence of IFN-α. It is showed that HCV had a strong effect on CD107a expression (C), especially on CD56dim subsets, but not IFN-γ production (D). [file Image_1.TIF]

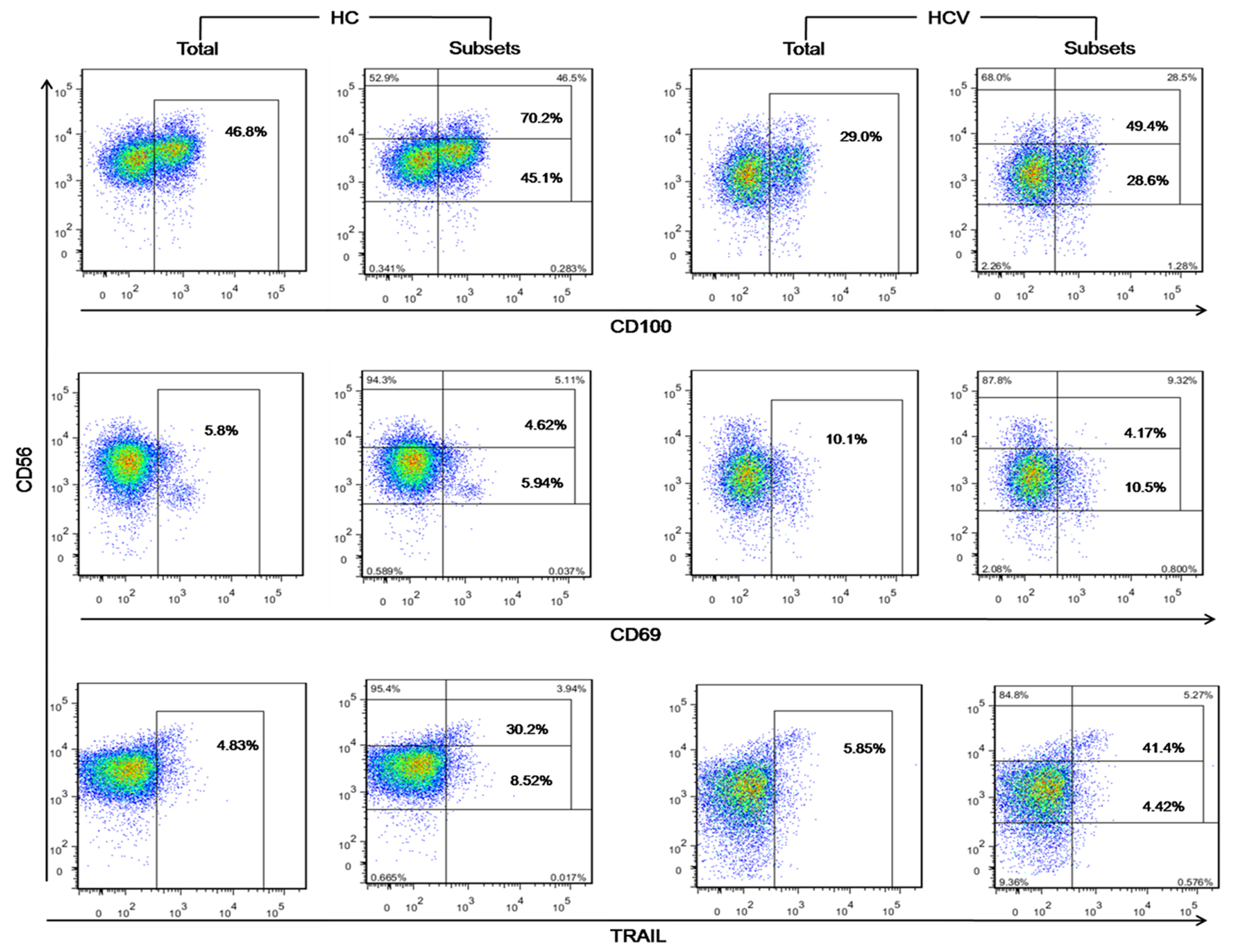

Supplement: Figure S2 — Frequency of CD100, CD69 and TRAIL expression on total NK and two subsets in patients with chronic HCV infection and healthy subjects, analyzed by flow cytometry. [file Image_2.TIF]

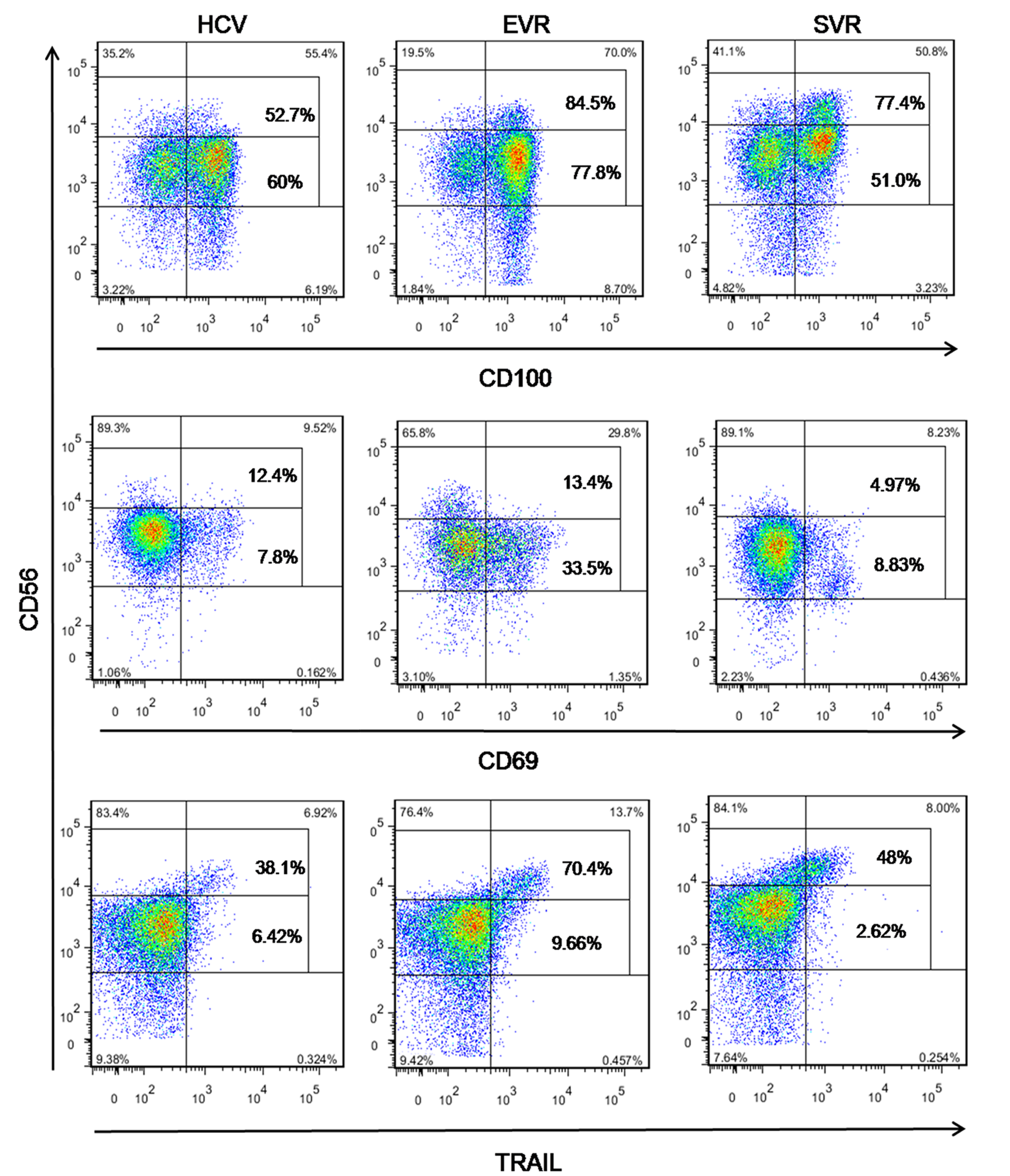

Supplement: Figure S3 — The percentage of CD100, CD69 and TRAIL expression on total NK and subsets in treatment-naive patients with chronic hepatitis C, patients with EVR and SVR after IFN-α-based therapy, analyzed by flow cytometry. [file Image_3.TIF]

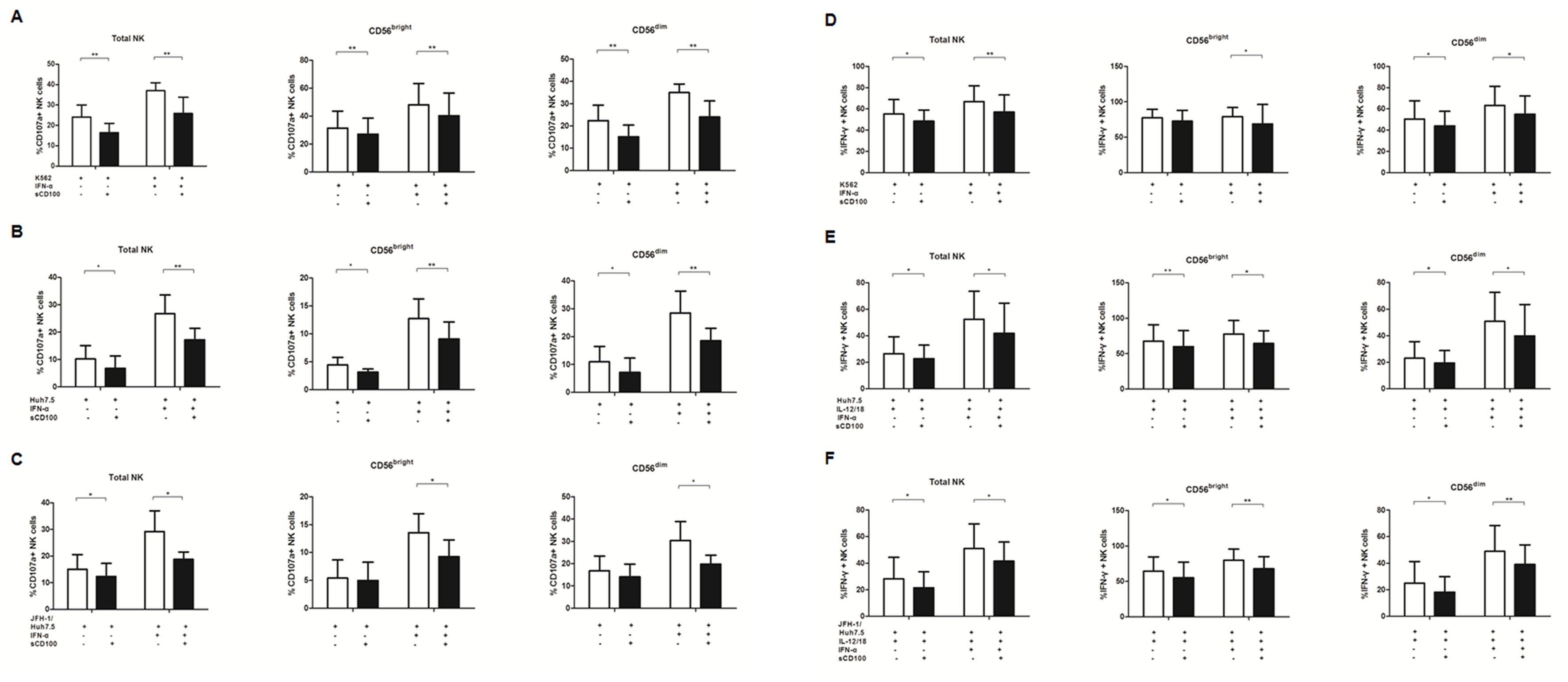

Supplement: Figure S4 — CD100-Plexin-B signaling influences NK cell functions. CD100-Plexin-B1/B2 interactions between NK and K562/Huh7.5 cells were blocked by sCD100 pre-incubating. CD107a and IFN-γ expressions were measured in total NK and the two subsets (CD56bright and CD56dim), in the presence or absence of IFN-α or sCD100. Paired Student t-test was used for the data analysis. [file Image_4.TIF]
