# Supplementary material for: Interferon-α-Enhanced CD100/Plexin-B1/B2 Interactions Promote Natural Killer Cell Functions in Patients with Chronic Hepatitis C Virus Infection
Source: Front Immunol. 2017 Nov 3;8:1435. doi: 10.3389/fimmu.2017.01435 (PMC5676449; doi:10.3389/fimmu.2017.01435)
Supplement: Supplementary file 5 [file Table_1.DOC]

Supplementary Table 1. Clinical characteristics of study subjects.

|  | HC  (n=24) | HCV  (n=30) | EVR  (n=25) | SVR  (n=20) |
| --- | --- | --- | --- | --- |
| Age[year mean±SEM] | 35.71±2.11 | 41.43±2.47 | 37.68±2.55 | 41.3±2.62 |
| Sex, Female/male | 12/12 | 15/15 | 12/13 | 10/10 |
| ALT, IU/ml[mean±SEM] | n.a | 88.17±15.9 | 33±4.68 | 23.31±3.71 |
| HCV-RNA mean log10cps/ml±SEM | n.a | 6.12±0.13 | <2.0 | <2.0 |
| HCV genotype 1b/2a | n.a | 13/13 | 10/12 | n.a |

Abbreviations: HC: healthy controls; HCV: treatment-naïve patients with chronic hepatitis C; EVR: HCV patients with EVR after 3-month antiviral treatment; SVR: patients with SVR after antiviral treatment; SEM: standard error of mean; n.a: not applicable. Note: HCV genotype was not identified in 4 and 3 patients in HCV and EVR groups, respectively.
