# Supplementary material for: Interferon-α-Enhanced CD100/Plexin-B1/B2 Interactions Promote Natural Killer Cell Functions in Patients with Chronic Hepatitis C Virus Infection
Source: Front Immunol. 2017 Nov 3;8:1435. doi: 10.3389/fimmu.2017.01435 (PMC5676449; doi:10.3389/fimmu.2017.01435)
Supplement: Supplementary file 6 [file Table_2.DOC]

Supplementary Table 2. Primer sequences used for realtime PCR analysis.

| Gene |  |  |
| --- | --- | --- |
| Symbol | Forward Primer (5’-3’) | Reverse Primer (5’-3’) |
| GAPDH | AGGTGAAGGTCGGAGTCAACG | AGGGGTCATTGATGGCAACA |
| Plexin-B1 | ACCAACTGCATTCACTCCCAA | GCACTCATCAGGCATCACAG |
| Plexin-B2 | TCCGGCAGGGTGCTATATGCT | GAAGGGCTTGTAGAAGATGTCAC |
